# Lactation duration and development of type 2 diabetes and metabolic syndrome in postpartum women with recent gestational diabetes mellitus

**DOI:** 10.1186/s13006-024-00632-1

**Published:** 2024-04-12

**Authors:** Sasiwan Suthasmalee, Chadakarn Phaloprakarn

**Affiliations:** 1 Women’s Health Center, MedPark Hospital, Bangkok, Thailand; 2 Department of Obstetrics and Gynecology, Faculty of Medicine Vajira Hospital, Navamindradhiraj University, 681 Samsen Road, Dusit District, Bangkok, 10300 Thailand

**Keywords:** Breastfeeding, Gestational diabetes mellitus, Lactation, Metabolic syndrome, Prediabetes, Postpartum, Type 2 diabetes

## Abstract

**Background:**

The World Health Organization and United Nations Children’s Fund recommend exclusive breastfeeding (EBF) for the first six months of an infant’s life. Although evidence suggests that maintaining breastfeeding has positive impacts on glucose and lipid metabolism in postpartum women with a history of gestational diabetes mellitus (GDM), no study has investigated whether such effects differ between breastfeeding intensities. This study aimed to evaluate the impact of maintaining breastfeeding on prediabetes, type 2 diabetes mellitus (T2DM), and metabolic syndrome (MetS) six months postpartum in women with GDM. This study also examined the potential variations in glucometabolic outcomes between EBF at six months and partial breastfeeding at six months.

**Methods:**

This prospective cohort study included 130 women with recent GDM who experienced live birth**s** between 7 September 2020 and 31 January 2023 at a university hospital in Bangkok, Thailand. All the women were free of T2DM and MetS at baseline (six weeks postpartum). We followed up these women six months postpartum to assess their breastfeeding practices (EBF at six months, partial breastfeeding at six months, or not maintaining breastfeeding) and evaluate their progression to prediabetes, T2DM, and MetS. Maintaining breastfeeding was defined as breastfeeding for six months. EBF was determined using the “recall since birth” method.

**Results:**

Of the 130 participants included, the rates of prediabetes, T2DM, and MetS six months postpartum were 33% (*n* = 43), 2% (*n* = 3), and 17% (*n* = 22), respectively. In the unadjusted model, maintaining breastfeeding was associated with a reduction in the risks of prediabetes and MetS but not T2DM. After adjusting for potential confounders, maintaining breastfeeding was a significant protective factor only for prediabetes. The adjusted risk ratios and 95% confidence intervals were 0.54 (0.29, 0.99) for prediabetes and 0.47 (0.19, 1.06) for MetS. When EBF at six months and partial breastfeeding at six months were separately analyzed, the risks of prediabetes and MetS differed between the two groups. In the EBF at six months-to-partial breastfeeding at six months comparison, the adjusted risk ratios (95% confidence intervals) of prediabetes and MetS were 0.46 (0.22, 0.97) vs. 0.79 (0.25, 2.49) and 0.34 (0.11, 0.99) vs. 0.69 (0.22, 2.07), respectively.

**Conclusions:**

Maintaining breastfeeding reduced the risk of prediabetes and MetS, but not of T2DM, six months postpartum; these effects were significant only with EBF. These findings indicate that supporting maternal efforts to practice EBF for six months may improve women’s health after GDM.

**Trial registration:**

Thai Clinical Trials Registry Registration No. TCTR20200902003. Date of registration: September 2, 2020. Date of initial participant enrollment: September 7, 2020.

## Background

The rising prevalence of gestational diabetes mellitus (GDM) globally has become an alarming issue recently [1, 2]. GDM has a significant impact not only on the outcomes of pregnancy [3, 4] but also on the risk of developing type 2 diabetes mellitus (T2DM) and metabolic syndrome (MetS) [5–8]. These elevated risks further contribute to a higher chance of developing cardiovascular diseases or mortality [9, 10].

Preventing T2DM and MetS is the key to enhancing the health of postpartum women with a history of GDM, and breastfeeding is the earliest prevention method after delivery. Women who breastfeed their babies for a longer period have been proven to be at reduced risk of developing T2DM, especially if they have experienced GDM [11–13]. However, little is known about the effect of lactation duration on preventing MetS [14]. In addition, these studies did not explore whether the effects were similar between exclusive breastfeeding (EBF) and partial breastfeeding.

Expert panels such as the World Health Organization (WHO) and United Nations Children’s Fund (UNICEF) recommend EBF for the first six months of an infant’s life [15]. Despite their recommendations, postpartum women with recent GDM tend to have a lower rate of EBF at hospital discharge and a shorter duration of EBF compared with women without GDM [16–18]. Therefore, further comprehensive studies with clear definitions of breastfeeding practices, including EBF, are needed to strengthen the evidence regarding the effect of maintaining breastfeeding, especially EBF, on the prevention of T2DM and MetS. This information can serve as substantial evidence to support women in their decision to exclusively breastfeed and help create a supportive environment for breastfeeding mothers.

This study aimed to determine the effects of maintaining breastfeeding on the risk of prediabetes, T2DM, and MetS six months postpartum in women with recent GDM. Furthermore, we investigated whether these outcomes differed between EBF at six months and partial breastfeeding at six months.

## Methods

### Setting and study design

This study was conducted as part of a study exploring the metabolic health of postpartum women with a history of GDM. This prospective cohort study was conducted at the Faculty of Medicine Vajira Hospital, Bangkok, Thailand. The study protocol was approved by the Vajira Institutional Review Board (certificate of approval no. 016/2563) and strictly adhered to the Strengthening the Reporting of Observational Studies in Epidemiology guidelines.

### Study population and eligibility criteria

The study participants were women with a GDM history who experienced live birth**s** between 7 September 2020 and 31 January 2023. To be included in this study, participants were required to be ≥ 18 years of age, have a pregnancy complicated by GDM diagnosed using the Carpenter and Coustan criteria [19], and have undergone blood glucose testing for GDM at our hospital. The exclusion criteria were HIV infection, pregnancy during the 6-month study period, diagnosis of T2DM or MetS at baseline (six weeks postpartum), taking medications or substances that are contraindicated during breastfeeding, such as chemotherapy agents, radioactive substances, illicit drugs, ergotamine, or lithium, and loss during follow-up.

### Sample size

Given that no study has directly investigated the effects of breastfeeding for six months on prediabetes, T2DM, and MetS six months postpartum in women with a history of GDM, we calculated the sample size based on data from a previous study that examined the effect of breastfeeding on glucose intolerance 12–14 months postpartum [20]. To show a 34% decrease in prediabetes/T2DM risk in women practicing breastfeeding for six months (from 78 to 44% [20]) with 80% power at a two-sided significance level of 0.05, at least 64 participants were required (32 who maintained breastfeeding and 32 who did not). Considering a dropout rate of 20%, the total sample size required was 80 (40 in each group).

### Participant recruitment and follow-up

In the postnatal ward, postpartum women who have been complicated by GDM in their most recent pregnancy (recent GDM) were recruited sequentially. They were informed about the research project and participated in the study. A written informed consent was obtained from all participants before enrollment. Baseline characteristics were extracted from the medical records. The pre-pregnancy body mass index (BMI) was calculated based on the self-reported pregravid weight and the measured height.

On the discharge date, the participants were provided with a mini calendar to record the date of introduction of infant formula or foods/drinks other than breast milk, as well as the date when the mother stopped breastfeeding or expressing milk. They were instructed to bring the calendar with them when they attended follow-up visits.

The participants were scheduled for follow-up visits at 6 weeks and 6 months postpartum. During both visits, the participants were interviewed about the frequency and duration of breastfeeding. In addition, they were asked whether they had provided infant formula or complementary foods to the babies. If so, the time of introduction of the formula or foods/drinks and the frequency of administration and quantity were recorded. Feeding practices were also assessed based on the data noted in the calendar provided to participants and the prospective assessment of breastfeeding status recorded in the hospital’s electronic database during the follow-up visits of the babies at the routine immunization clinics at the completion of the 2nd, 4th, and 6th months after birth. Maternal weight, waist circumference (WC), and blood pressure (BP) were measured by a specially trained nurse using standardized protocols. Venous blood samples were collected at both visits. At six weeks postpartum, blood samples were collected to measure fasting plasma glucose (FPG), fasting lipids such as total cholesterol, triglycerides (TG), low-density lipoprotein cholesterol, high-density lipoprotein cholesterol (HDL-C), and plasma glucose levels 2 h after consuming a 75-g oral glucose load. At six months postpartum, fasting venous blood samples were collected to measure FPG, hemoglobin A1c (HbA1c), and lipid parameters.

### Laboratory measurements

Blood samples were collected in the morning after fasting for 12 h overnight. Blood tests for plasma glucose, HbA1c, and lipid levels were performed using standard assays with a well-calibrated analyzer. Our laboratory received approval from the Randox International Quality Assessment Scheme for blood chemistry and HbA1c analyses. The HbA1c test was performed using a standardized and certified assay by the National Glycohemoglobin Standardization Program. This assay can be traced to the reference method used in the Diabetes Control and Complications Trial, thereby ensuring accurate and reliable results.

### Outcome measures and definitions

The outcome measures were prediabetes, T2DM, and MetS. The exposures of interest were lactation duration and intensity. Those with FPG levels of 100–125 mg/dL, 2-h plasma glucose levels of 140–199 mg/dL, or HbA1c levels of 5.7–6.4% were diagnosed with prediabetes [21]. T2DM was defined as FPG ≥ 126 mg/dL, 2-h plasma glucose during a 75-g oral glucose tolerance test ≥ 200 mg/dL, or HbA1c ≥ 6.5% [21]. MetS was diagnosed based on a Joint Interim Statement [22]. A diagnosis was made when three or more of the following were present: (1) WC ≥ 80 cm, (2) systolic BP ≥ 130 or diastolic BP ≥ 85 mmHg or treatment with antihypertensive medication, (3) FPG ≥ 100 mg/dL or treatment with diabetes medication, (4) fasting TG ≥ 150 mg/dL or medication treatment, and (5) HDL-C < 50 mg/dL or medication treatment.

Because this study limited the time for glucometabolic evaluation to six months postpartum, we categorized the lactation duration as six months (maintaining breastfeeding) or less than six months (not maintaining breastfeeding). Given that the WHO and UNICEF recommend that mothers exclusively breastfeed their infants for six months, the intensity of maintaining breastfeeding was divided into EBF and partial breastfeeding. We defined EBF at six months (maintaining EBF) as feeding infants only breast milk for six months, except for drops or syrups containing medicines, vitamins, or mineral supplements. When infants received any other food or liquid during the 6-month breastfeeding period, they were categorized as partial breastfeeding at six months.

### Statistical analysis

Data were analyzed using IBM SPSS Statistics for Windows, Version 28.0 (IBM Corp., Armonk, NY, USA). Categorical variables are presented as numbers and percentages and were compared using the chi-squared or Fisher’s exact tests. Continuous variables were described as means and standard deviations for normally distributed data or as medians and interquartile ranges for non-normally distributed data and were compared using the Student’s *t*-test or Mann–Whitney U test. To estimate the associations of lactation duration with prediabetes, T2DM, and MetS, generalized linear models were used to control for potential confounders, which were determined a priori based on established associations in a previous study [20] and were limited to age, family history of diabetes, pre-pregnancy BMI, weight change from delivery to six months postpartum, and prediabetes at baseline. The results are reported as risk ratios (RRs) with 95% confidence intervals (CIs). Statistical significance was set at *p* < 0.05.

## Results

### Participant characteristics

**Fig. 1:**
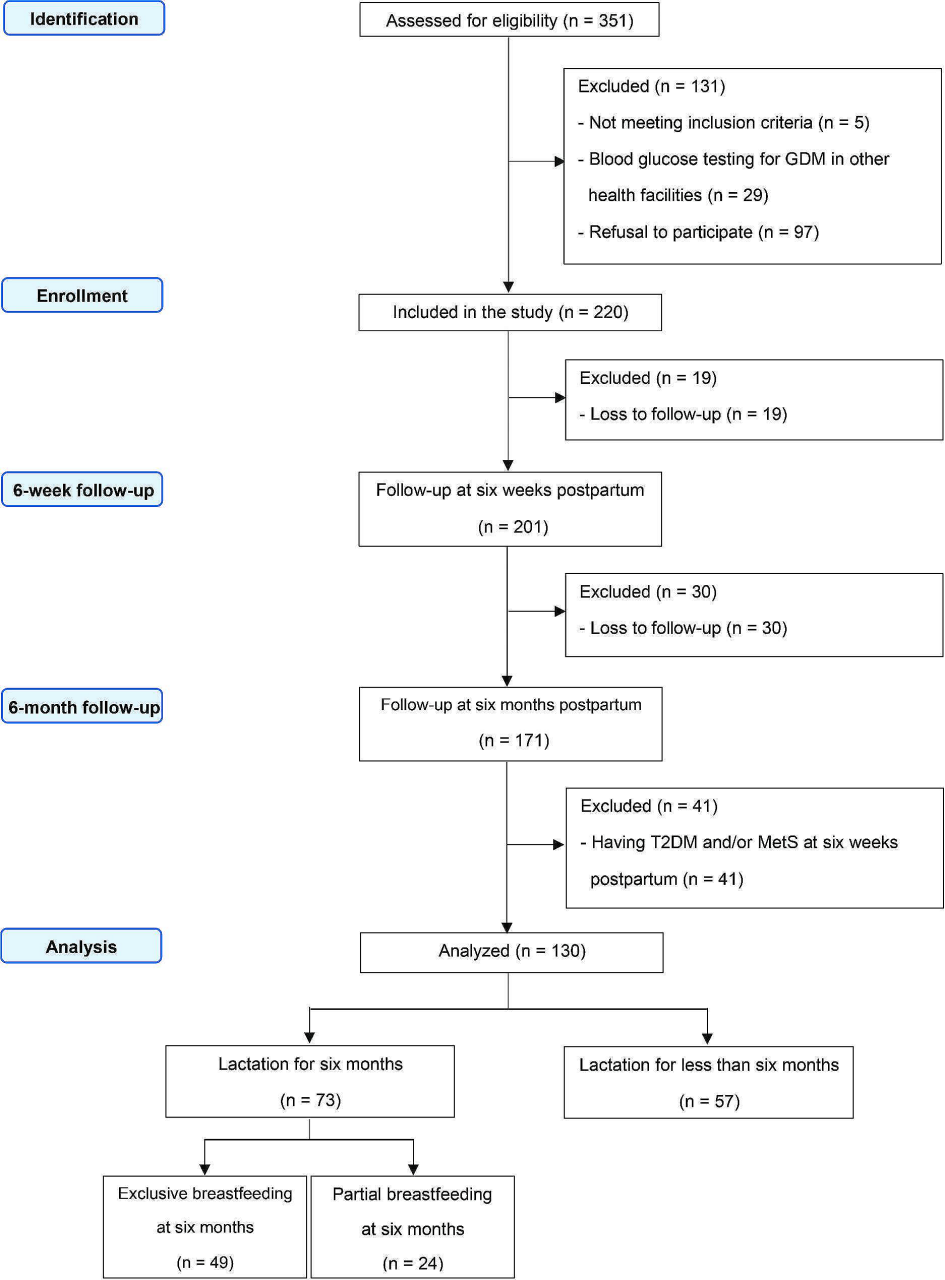
STROBE flow chart. *Abbreviation* *GDM* gestational diabetes mellitus, *MetS* metabolic syndrome, *STROBE* Strengthening the Reporting of Observational Studies in Epidemiology, *T2DM* type 2 diabetes mellitus

The flow diagram illustrating the participant selection process is shown in Fig. 1. Of the 220 women enrolled, 90 were excluded owing to loss during follow-up (*n* = 49) or diagnosis of T2DM and/or MetS at baseline (*n* = 41). The final sample comprised 130 women. The characteristics at prenatal visits and delivery of the women who were included and those excluded were not different. Among the 130 women included, 73 (56.2%) women breastfed for six months (maintaining breastfeeding) and 57 (43.8%) breastfed for less than six months (not maintaining breastfeeding).

Table 1 summarizes the participant characteristics. No significant difference in the characteristics during prenatal visits or delivery was observed between participants who maintained breastfeeding and those who did not. At six weeks postpartum, participants in both groups had similar prediabetes rates (21.9% and 24.6%, respectively; *p* = 0.723). Compared with participants who did not maintain breastfeeding, those who maintained breastfeeding had significantly lower FPG and TG levels but significantly higher levels of total cholesterol and HDL-C at 6 weeks and 6 months postpartum. From delivery to six months postpartum, participants who maintained breastfeeding lost almost 2 kg more weight than those who did not (10.4 kg vs. 8.7 kg, *p* = 0.039).

**Table 1:** Characteristics of the participants by lactation duration group

Characteristic	Breastfeeding for six months	Breastfeeding for less than six months	p-value
	(*n* = 57)	(*n* = 73)	
**At prenatal visits**			
Age (years)	32.2 ± 6.0	33.6 ± 5.2	0.176
Pre-pregnancy weight (kg)	58.0 (50.1–78.0)	56.6 (52.0–66.0)	0.201
Pre-pregnancy BMI (kg/m^2^)	23.9 (20.5–30.2)	23.7 (21.3–26.9)	0.743
Parity			0.474
Primiparous	27 (47.4)	30 (41.1)	
Multiparous	30 (52.6)	43 (58.9)	
Family history of diabetes	22 (38.6)	22 (30.1)	0.312
Insulin use during pregnancy	4 (7.0)	6 (8.2)	0.799
Gestational weight gain (kg)	10.3 ± 5.2	10.8 ± 5.8	0.637
**At delivery**			
Weight (kg)	72.2 (62.8–88.3)	69.4 (64.4–78.2)	0.225
Gestational age at delivery (weeks)	38.0 (37.0–38.5)	38.0 (38.0–39.0)	0.328
Mode of delivery			0.669
Vaginal	31 (54.4)	41 (56.2)	
Cesarean	26 (45.6)	32 (43.8)	
**At six weeks postpartum**			
Weight (kg)	62.0 (53.6–75.6)	59.0 (53.5–66.1)	0.341
BMI (kg/m^2^)	24.3 (21.0–28.8)	24.0 (22.3–26.2)	0.680
Weight change from delivery to six weeks postpartum (kg)	−10.2 ± 3.7	−10.3 ± 2.6	0.858
WC (cm)	82.9 ± 10.8	82.9 ± 10.5	0.989
Systolic BP (mmHg)	118.0 ± 13.8	118.3 ± 10.1	0.883
Diastolic BP (mmHg)	72.3 ± 8.6	73.0 ± 9.2	0.629
FPG (mg/dL)	89.9 ± 6.2	87.1 ± 7.5	0.027
2-h PG (mg/dL)	112.0 (98.0–142.5)	121.0 (100.0–145.0)	0.168
TC (mg/dL)	208.0 (187.5–226.0)	220.0 (191.5–253.5)	0.042
TG (mg/dL)	107.0 (83.5–150.0)	89.0 (65.0–107.0)	< 0.001
LDL-C (mg/dL)	142.0 (118.5–158.0)	148.0 (116.5–176.5)	0.163
HDL-C (mg/dL)	55.0 (50.5–64.5)	66.0 (57.0–77.5)	< 0.001
**At six months postpartum**			
Weight (kg)	61.7 (52.7–76.8)	58.7 (52.4–67.3)	0.095
BMI (kg/m^2^)	24.5 (21.7–29.6)	24.1 (21.6–26.1)	0.293
Weight change from delivery to six months postpartum (kg)	−8.7 ± 4.5	−10.4 ± 4.5	0.039
WC (cm)	82.7 ± 11.7	80.9 ± 11.3	0.386
Systolic BP (mmHg)	117.0 ± 11.7	118.2 ± 9.6	0.523
Diastolic BP (mmHg)	71.6 ± 10.2	73.6 ± 9.6	0.251
FPG (mg/dL)	96.0 (90.5–103.5)	94.0 (88.0–97.0)	0.037
HbA1c (%)	5.5 (5.2–5.7)	5.4 (5.2–5.6)	0.277
TC (mg/dL)	190.0 (175.0–205.5)	204.0 (187.0–230.0)	0.004
TG (mg/dL)	100.0 (73.5–149.0)	67.0 (54.0–99.5)	< 0.001
LDL-C (mg/dL)	125.0 (110.0–139.5)	135.0 (116.0–160.5)	0.035
HDL-C (mg/dL)	52.0 (45.0–63.5)	65.0 (53.0–77.5)	< 0.001

Data are presented as the mean ± SD or median (IQR) or n (%)

*Abbreviations* *BMI* body mass index, *BP* blood pressure, *FPG* fasting plasma glucose, *HbA1c* hemoglobin A1c, *HDL-C* high-density lipoprotein cholesterol, *IQR* interquartile range, *LDL-C* low-density lipoprotein cholesterol, *PG* postprandial glucose, *SD* standard deviation, *TC* total cholesterol, *TG* triglycerides, *WC* waist circumference

### Effects of lactation duration on prediabetes, T2DM, and MetS

Of the 130 participants, the rates of prediabetes, T2DM, and MetS six months postpartum were 33.1% (*n* = 43), 2.3% (*n* = 3), and 16.9% (*n* = 22), respectively (Table 2). Participants who maintained breastfeeding were less likely than those who did not to have prediabetes or MetS six months postpartum: 24.7% vs. 43.9%, *p* = 0.021 for prediabetes and 11.0% vs. 24.6%, *p* = 0.040 for MetS. Participants in both groups showed no significant difference in T2DM rates.

**Table 2:** Unadjusted and adjusted risk ratios of prediabetes, type 2 diabetes mellitus, and metabolic syndrome in the maintaining breastfeeding group

	Not maintaining breastfeeding^a^	Maintaining breastfeeding	Unadjusted RR (95% CI)	Adjusted RR (95% CI)
	(*n* = 57)	(*n* = 73)		
Prediabetes	25 (43.9)	18 (24.7)	0.56 (0.34, 0.92)	0.54 (0.29, 0.99)^b^
T2DM	1 (1.8)	2 (2.7)	1.56 (0.15, 16.80)	–
MetS	14 (24.6)	8 (11.0)	0.45 (0.20, 0.99)	0.47 (0.19, 1.06)^c^

Data are presented as n (%), unless otherwise specified

*Abbreviations* *BMI* body mass index, *CI* confidence interval, *MetS* metabolic syndrome, *RR* risk ratio, *T2DM* type 2 diabetes mellitus

^a^ Reference group

^b^ Adjusted for age, family history of diabetes, pre-pregnancy BMI, weight change from delivery to six months postpartum, and prediabetes at baseline

^c^ Adjusted for age, pre-pregnancy BMI, weight change from delivery to six months postpartum, and prediabetes at baseline

**Table 3:** Unadjusted and adjusted risk ratios of prediabetes and metabolic syndrome six months postpartum in participants with exclusive breastfeeding at six months and partial breastfeeding at six months

	Breastfeeding practice group					
	**Not maintaining breastfeeding** (*n* = 57)	**Partial breastfeeding at six months** (*n* = 24)		**Exclusive breastfeeding at six months** (*n* = 49)		
		**Unadjusted RR (95% CI)**	**Adjusted RR (95% CI)**	**Unadjusted RR (95% CI)**	**Adjusted RR (95% CI)**	
Prediabetes	1.00 (reference)	0.76 (0.40, 1.44)	0.79 (0.25, 2.49)^a^	0.47 (0.25, 0.87)	0.46 (0.22, 0.97)^a^	
MetS	1.00 (reference)	0.68 (0.25, 1.85)	0.69 (0.22, 2.07)^b^	0.33 (0.12, 0.94)	0.34 (0.11, 0.99)^b^	

*Abbreviations* *BMI* body mass index, *CI* confidence interval, *MetS* metabolic syndrome, *RR* risk ratio

^a^Adjusted for age, family history of diabetes, pre-pregnancy BMI, weight change from delivery to six months postpartum, and prediabetes at baseline

^b^Adjusted for age, pre-pregnancy BMI, weight change from delivery to six months postpartum, and prediabetes at baseline

In the unadjusted model, maintaining breastfeeding was associated with a reduction in the risk of prediabetes and MetS. After adjusting for potential confounders, maintaining breastfeeding was a significant protective factor only for prediabetes. The adjusted RRs and 95% CIs were 0.54 (0.29, 0.99) for prediabetes and 0.47 (0.19, 1.06) for MetS.

**Fig. 2:**
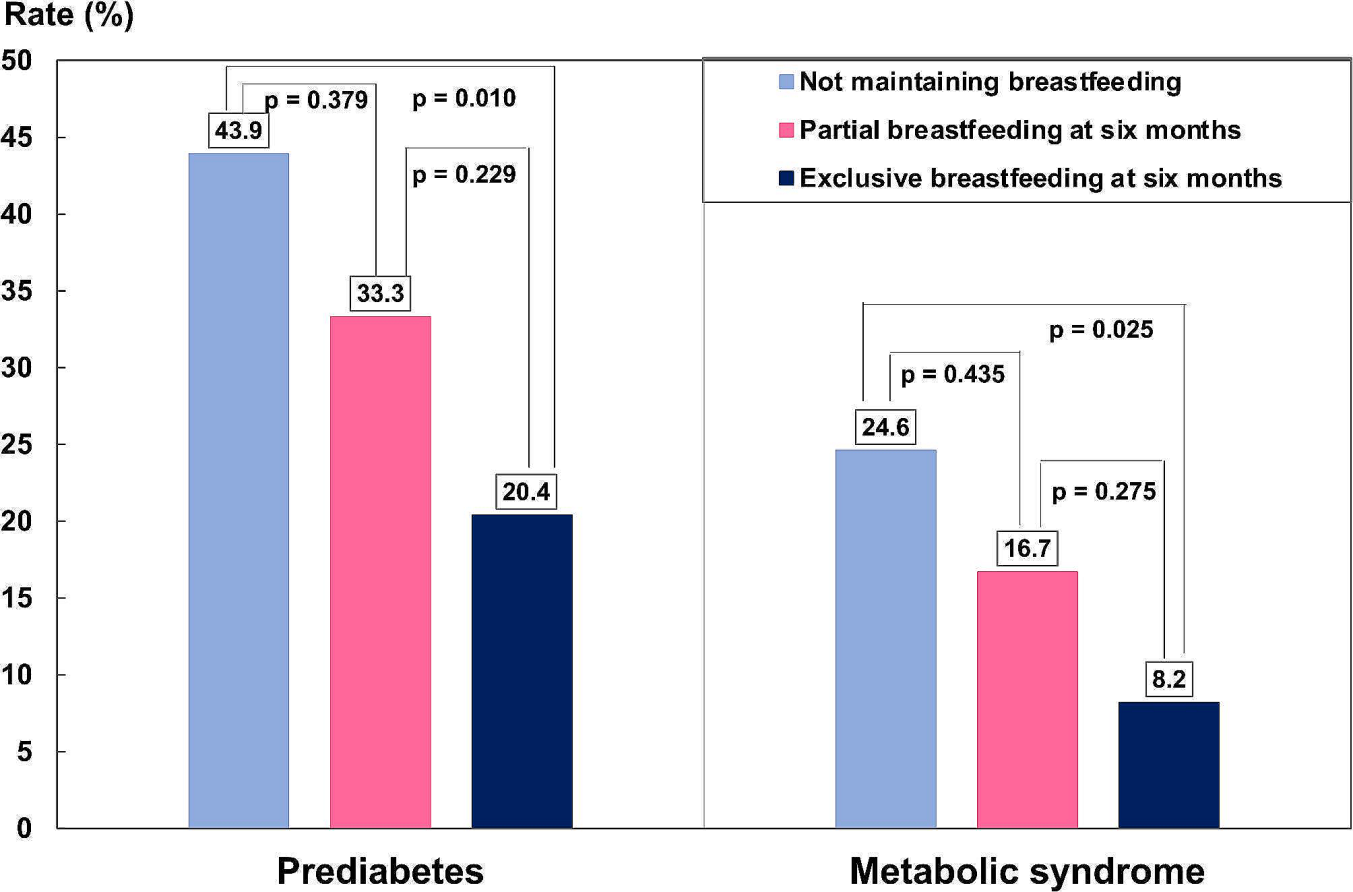
Rates of prediabetes and metabolic syndrome six months postpartum by the breastfeeding practice group

To determine whether the effects of maintaining breastfeeding on prediabetes and MetS differed between breastfeeding intensities, we divided the 73 participants who maintained breastfeeding into two separate groups: EBF at six months (*n* = 49) and partial breastfeeding at six months (*n* = 24). Figure 2 shows that participants who exclusively breastfed at six months had the lowest rates of prediabetes and MetS six months postpartum, followed by participants who were partially breastfeeding at six months and participants who did not maintain breastfeeding. In the adjusted models (Table 3), the protective effects of maintaining breastfeeding against prediabetes and MetS were significant only in the EBF group. In the EBF at six months-to-partial breastfeeding at six months comparison, the adjusted RRs (95% CIs) of prediabetes and MetS were 0.46 (0.22, 0.97) vs. 0.79 (0.25, 2.49) and 0.34 (0.11, 0.99) vs. 0.69 (0.22, 2.07), respectively.

## Discussion

The main findings of this study were that maintaining EBF for up to six months was associated with risk reductions in both prediabetes and MetS at six months postpartum. Specifically, the risk reduction for prediabetes was 54%, while the risk reduction for MetS was higher at 66%.

Several studies have demonstrated an inverse association between lactation duration and T2DM risk long-term after delivery. A study involving 1,010 women who participated in the Study of Women, Infant Feeding, and Type 2 Diabetes after GDM revealed that breastfeeding for more than 10 months resulted in a 53% reduction in T2DM occurrence two years after delivery [11]. Another study that included 304 women with prior GDM reported the protective effect (hazard ratio 0.54; 95% CI 0.34, 0.85) of breastfeeding for more than three months against the development of T2DM for approximately 19 years after delivery in islet autoantibody-negative women [12]. In another 25-year follow-up study involving 4,372 women with a GDM history, EBF for > 6 to 12 months was associated with a 21% decrease in T2DM risk [13].

In contrast to previous studies, this study investigated the occurrence of T2DM over a shorter follow-up period. No protective association between maintaining breastfeeding and the development of T2DM was found at six months postpartum. This lack of association may be owing to the small number of incident cases, which may have limited our ability to assess the differences between the maintaining and not maintaining breastfeeding groups. Another reason may be that GDM usually progresses to prediabetes, which is a precursor to T2DM diagnosis, a few years after delivery [23]. Hence, the early effect of breastfeeding on glucose metabolism is likely to manifest as a decrease in prediabetes occurrence rather than T2DM. This hypothesis was confirmed by the findings of this study, which showed that breastfeeding, particularly EBF, for six months significantly reduced the risk of prediabetes six months postpartum. Consistent with the results of this study, Yasuhi et al. reported that intensive breastfeeding for at least six months had a protective effect against the development of glucose intolerance during the first year postpartum [20]. Although this study focused on lactation duration, whereas Yasuhi et al. focused on lactation intensity, the concordant findings of both studies underscore the importance of combining longer duration and higher intensity of breastfeeding, rather than longer duration or higher intensity of breastfeeding alone, as a preventive measure against postpartum glucose intolerance after GDM.

In addition to prediabetes, the results of this study showed that maintaining EBF but not maintaining partial breastfeeding reduced the risk of MetS six months postpartum. These findings suggested that a lack of EBF at six months was associated with a higher risk of MetS. In women who completed six months of EBF, the risk reduction for incident MetS was 66%. This estimate was independent of key potential confounders, including age, BMI, postpartum weight gain, and prediabetes at baseline. The results of this study are different from the findings of Gunderson et al. [14], who enrolled 84 women with prior GDM in the Coronary Artery Risk Development in Young Adults (CARDIA) study and found a significant association between any breastfeeding (either EBF or partial breastfeeding) for a longer duration and reduced risk of MetS > 20 years of follow-up. The different findings between the two studies may be owing to the differences in sample size, the time point at which MetS was assessed, and the criteria used to define MetS. Gunderson et al. used the NCEP ATP III criteria for diagnosing MetS [14], whereas our study defined MetS based on the Joint Interim Statement definition.

However, the underlying mechanisms by which maintaining lactation improves glucose and lipid homeostasis remain unclear. A longer lactation duration may enhance weight loss, resulting in improved metabolic parameters [14]. In this study, we observed greater postpartum weight loss in participants who maintained breastfeeding than those who did not. However, other mechanisms may also be involved because we identified EBF at six months as an independent protective factor for prediabetes and MetS after adjusting for postpartum weight change. In experimental models, prolactin (PRL), a pituitary hormone responsible for milk production, improves glucose metabolism via pancreatic beta cell upregulation and increases insulin secretion [24, 25]. In human adipose tissue, PRL decreases lipogenesis by suppressing malonyl-CoA concentration [26]. Given the essential role of the adipose tissue in maintaining glucose and lipid homeostasis [27], PRL may mediate the association between maintaining breastfeeding and lower risks of prediabetes and MetS.

The findings of this study have clinical implications for preventing glucose and lipid metabolic abnormalities after GDM. Our results showed that EBF for six months reduced the risk of prediabetes and MetS by 54% and 66%, respectively, at six months postpartum. Therefore, we strongly support the recommendations of the WHO and UNICEF, which state that postpartum women should be supported in achieving EBF for the first six months of a baby’s life. This is particularly important for women with pregnancies complicated by GDM who are at risk of developing glucometabolic disorders and subsequent cardiovascular diseases. Likewise, the findings of this study can be used as solid evidence to amplify the public health message about the importance of EBF and support women in achieving this goal.

This study had several strengths. First, the prospective cohort design ensured the systematic determination of breastfeeding practices and glucose and lipid parameters in all participants at 6 weeks and 6 months postpartum. Second, we included women who were T2DM- and MetS-free at baseline to guarantee that the diagnoses of both conditions six months postpartum were new. Third, the measurements of weight, WC, and BP were performed using standardized criteria to ensure the precision of the assessment. Finally, this study adds new data, revealing that the effects of longer lactation duration on glucose and lipid metabolism differ between different breastfeeding intensities.

Despite its strengths, the study also had some limitations. First, owing to the small number of T2DM cases six months postpartum, this study was underpowered to detect differences in T2DM risk between the two lactation duration groups. Second, the findings of this study may be subject to selection bias because the participants who completed the 6-month study may have differed from those who did not. However, this effect was considered negligible because we did not observe any differences in the characteristics of the two groups of participants. Third, owing to the limited number of participants, we did not exclude individuals with prediabetes at baseline. This may have affected the occurrence of prediabetes six months postpartum. Nevertheless, the rates of prediabetes at baseline did not differ between participants who maintained breastfeeding and those who did not. Additionally, we incorporated prediabetes at baseline as a potential confounding variable in the generalized linear model. Hence, the effect of maintaining breastfeeding on prediabetes occurrence six months postpartum was independent of this variable. Fourth, assessing breastfeeding practices based on the interviews at 6 weeks and 6 months postpartum may have low validity owing to recall bias. Nevertheless, we attempted to reduce this effect by using other methods (prospective data recording in a calendar and the hospital’s electronic database during the baby’s routine follow-up visits) to improve the quality of breastfeeding data. Finally, this study’s findings must be interpreted with the understanding that all participants were Asian, and a diagnosis of MetS was made using the Joint Interim Statement definition. Therefore, the generalizability of our results must be verified in other population groups or settings in which different diagnostic criteria for MetS are used.

## Conclusions

Overall, maintaining EBF for up to six months was found to reduce the risk of prediabetes and MetS, but not T2DM, at six months postpartum. These data indicate that supporting maternal breastfeeding efforts may be an important way to improve women’s health after GDM. In addition, taking a history of breastfeeding practices from parous women may aid health professionals in identifying and closely monitoring women with a history of shorter-duration or lower-intensity breastfeeding who are at risk of developing glucose and lipid metabolism abnormalities.

## Data Availability

The datasets used and analyzed in this study are available from the corresponding author upon reasonable request.

## Abbreviations

BP: Blood pressure
BMI: Body mass index
CI: Confidence interval
EBF: Exclusive breastfeeding
FPG: Fasting plasma glucose
GDM: Gestational diabetes mellitus
HbA1c: Hemoglobin A1c
HDL-C: High-density lipoprotein cholesterol
MetS: Metabolic syndrome
PRL: Prolactin
RR: Risk ratio
T2DM: Type 2 diabetes mellitus
TG: Triglycerides
UNICEF: United Nations Children's Fund
WC: Waist circumference
WHO: World Health Organization
