# Correction: Lactation duration and development of type 2 diabetes and metabolic syndrome in postpartum women with recent gestational diabetes mellitus

**DOI:** 10.1186/s13006-024-00640-1

**Published:** 2024-04-29

**Authors:** Sasiwan Suthasmalee, Chadakarn Phaloprakarn

**Affiliations:** 1 Women’s Health Center, MedPark Hospital, Bangkok, Thailand; 2 Department of Obstetrics and Gynecology, Faculty of Medicine Vajira Hospital, Navamindradhiraj University, 681 Samsen Road, Dusit District, Bangkok, 10300 Thailand

**International Breastfeeding Journal (2024) 19:25**

10.1186/s13006-024-00632-1

Following publication of the article, it came to the authors’ attention that the author list and the journal name information detailed in reference number 4 were incorrect. The reference has since been corrected; please refer to the current version of the reference. The authors thank you for reading and apologize for any inconvenience caused.

